# Recent Advances in Non-Viral Vectors for Gene Therapy and Gene Delivery: From Lipid Nanoparticles to Engineered Extracellular Vesicles

**DOI:** 10.3390/pharmaceutics18091094

**Published:** 2026-08-30

**Authors:** Yongfeng Yang, Tingting Song, Kaili Huang, Hong Huang, Maoyuan Zhao, Yi Li

**Affiliations:** 1 Institute of Respiratory Health and Multimorbidity, Department of Pulmonary and Critical Care Medicine, West China Hospital, Sichuan University, Chengdu 610041, China; yangyongfeng0523@163.com (Y.Y.); songtt9292@gmail.com (T.S.); jon0824hh@163.com (H.H.); 2 Department of Thoracic Surgery, Lung Cancer Center/Lung Cancer Institute, West China Hospital, Sichuan University, Chengdu 610041, China; 18380381429@163.com; 3 Lung Cancer Center/Lung Institute & State Key Laboratory of Respiratory Health and Multimorbidity, Department of Medical Oncology, Cancer Center, West China Hospital, Sichuan University, Chengdu 610041, China

**Keywords:** non-viral vectors, gene therapy, gene delivery, lipid nanoparticles, polymeric nanoparticles, extracellular vesicles, CRISPR, genome editing, mRNA delivery

## Abstract

Gene therapy and genome editing increasingly depend on the safe, effective, and cell-selective delivery of nucleic acids and protein–nucleic acid complexes. Although viral vectors remain important for applications requiring durable gene expression, non-viral vectors offer advantages in cargo capacity, modularity, transient expression, potential repeat dosing, and avoidance of vector–genome integration. Lipid nanoparticles (LNPs), polymeric nanoparticles, inorganic nanomaterials, extracellular vesicles (EVs), and biomimetic hybrid systems have consequently become central platforms for delivery of siRNA, mRNA, plasmid DNA, antisense oligonucleotides, and CRISPR-based genome editors. Among these, ionizable LNPs are currently the most clinically mature non-viral technology, supported by the clinical success of siRNA therapeutics and mRNA vaccines, as well as the emergence of in vivo CRISPR therapies. Nevertheless, efficient endosomal escape, cell-type-selective targeting, extrahepatic delivery, and repeat-dose tolerability remain substantial barriers. Polymeric vectors provide broad chemical tunability, allowing adjustment of charge density, degradability, stimulus responsiveness, intracellular trafficking, and cargo release. However, toxicity and batch-to-batch reproducibility remain key concerns. EVs provide a biologically derived alternative with favorable membrane interfaces and potential advantages for protein and ribonucleoprotein delivery, but their clinical translation is constrained by heterogeneity, loading efficiency, product characterization, and scalable manufacturing. This review summarizes recent advances in non-viral gene-delivery platforms, compares their strengths and limitations, and discusses future directions in cell-selective delivery, endosomal escape, transient delivery of genome-editing machinery, engineered EVs, hybrid vectors, and manufacturing-oriented development. The field is transitioning from organ-level delivery toward delivery of the correct payload to the correct cell type at a clinically relevant exposure and safety margin.

## 1. Introduction

Gene therapy has evolved beyond the delivery of replacement DNA into a broad therapeutic landscape including siRNA, antisense oligonucleotides, mRNA, plasmid DNA, protein therapeutics, CRISPR/Cas nucleases, base editors, and prime editors. The therapeutic promise of these modalities is constrained not only by the biological activity of the cargo but also by whether it can be delivered to the appropriate cells, released into the correct intracellular compartment, and expressed or functionally active for an appropriate duration [1,2,3,4,5,6,7,8].

The required delivery strategy differs substantially by cargo type (Table 1). Plasmid DNA must enter the nucleus before transcription, whereas siRNA and mRNA primarily require cytosolic release. Cas9 mRNA/sgRNA systems require coordinated intracellular translation and RNP assembly, while preassembled Cas RNPs require preservation of protein activity, co-delivery of guide RNA, cytosolic release, and nuclear access. These distinctions explain why no single non-viral carrier is universally optimal across all nucleic-acid modalities [5,6,7,8,9,10].

Viral vectors, including adeno-associated virus, adenovirus, and lentivirus, offer high transduction efficiency and can provide durable expression. However, limitations include restricted packaging capacity, pre-existing immunity, vector-induced immunogenicity, difficult repeat dosing, manufacturing complexity, and—in some contexts—concerns about persistent nuclease expression or insertional mutagenesis [1,2,3,4,5,6,7]. Non-viral vectors are attractive alternatives because they are generally modular, can accommodate diverse cargoes, often support transient activity, and can be designed to degrade after cargo release [8,9,10,11,12,13,14,15].

**Figure 1:**
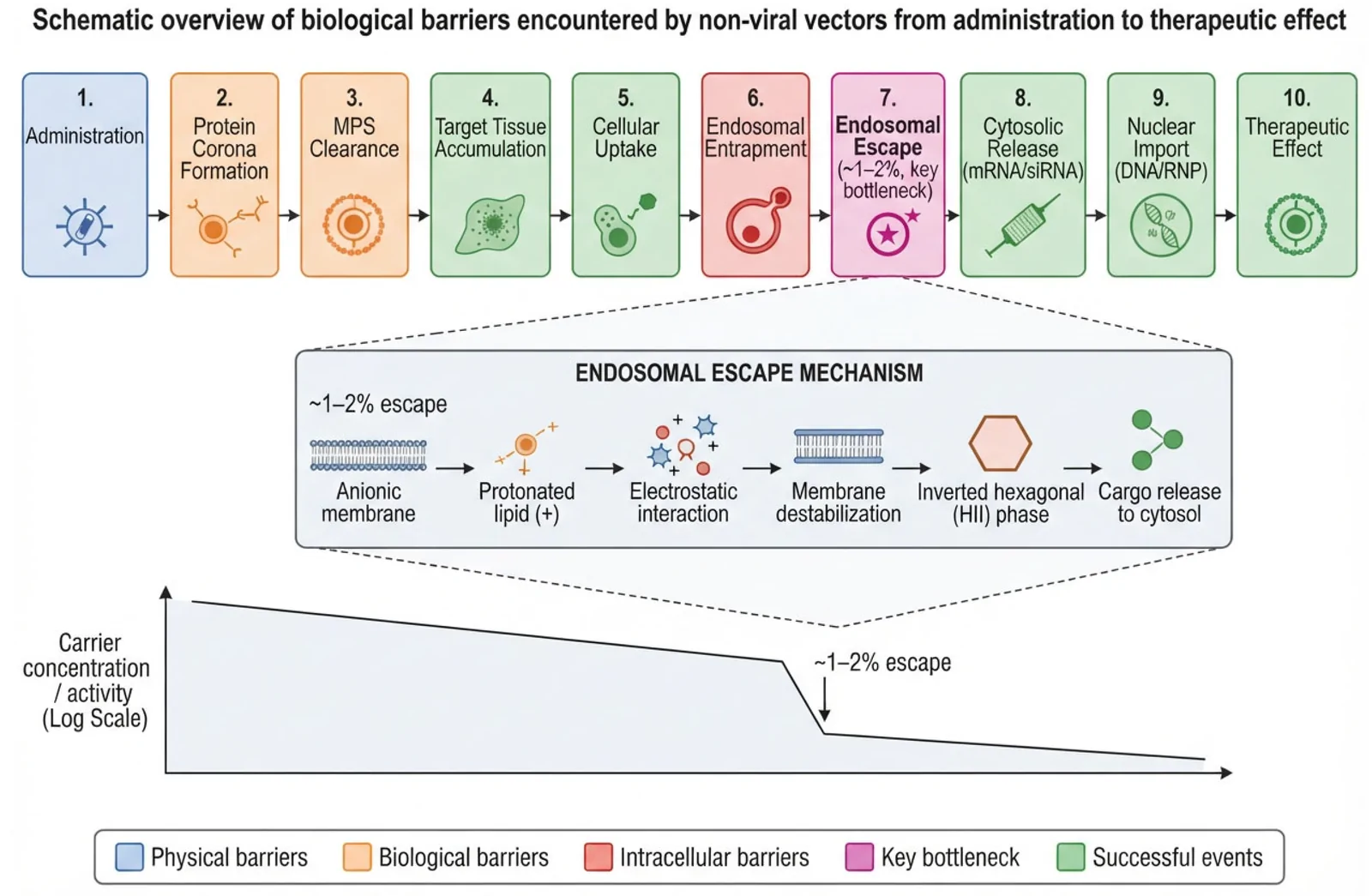
Schematic overview of biological barriers encountered by non-viral vectors from administration to therapeutic effect. Following administration, vectors encounter a sequential series of barriers: protein corona formation in the circulation, clearance by the mononuclear phagocyte system (MPS) in the liver and spleen, target tissue accumulation and extravasation, cellular uptake via endocytosis, and endosomal entrapment. Endosomal escape represents the major quantitative bottleneck, with only approximately 1–2% of internalized cargo successfully reaching the cytosol. For plasmid DNA and RNP cargoes, additional nuclear import is required. The lower panel illustrates the progressive decline in carrier concentration and functional activity across these barriers. Color coding: blue, physical barriers; orange, biological barriers; red, intracellular barriers; magenta, key bottleneck; green, successful delivery events.

However, non-viral delivery is a multistage problem. After administration, vectors may encounter nuclease degradation, protein-corona formation, mononuclear phagocyte system uptake, renal or hepatobiliary clearance, vascular barriers, extracellular matrix barriers, unproductive endocytosis, endosomal sequestration, poor cytosolic release, and inefficient nuclear import of DNA or genome editors. It is estimated that of the nanoparticles internalized by cells, only approximately 1–2% successfully escape endosomes to reach the cytosol [15,16,17,18]. Therefore, apparent nanoparticle uptake should not be confused with productive intracellular delivery. This multi-step barrier cascade is illustrated schematically in Figure 1.

**Figure 2:**
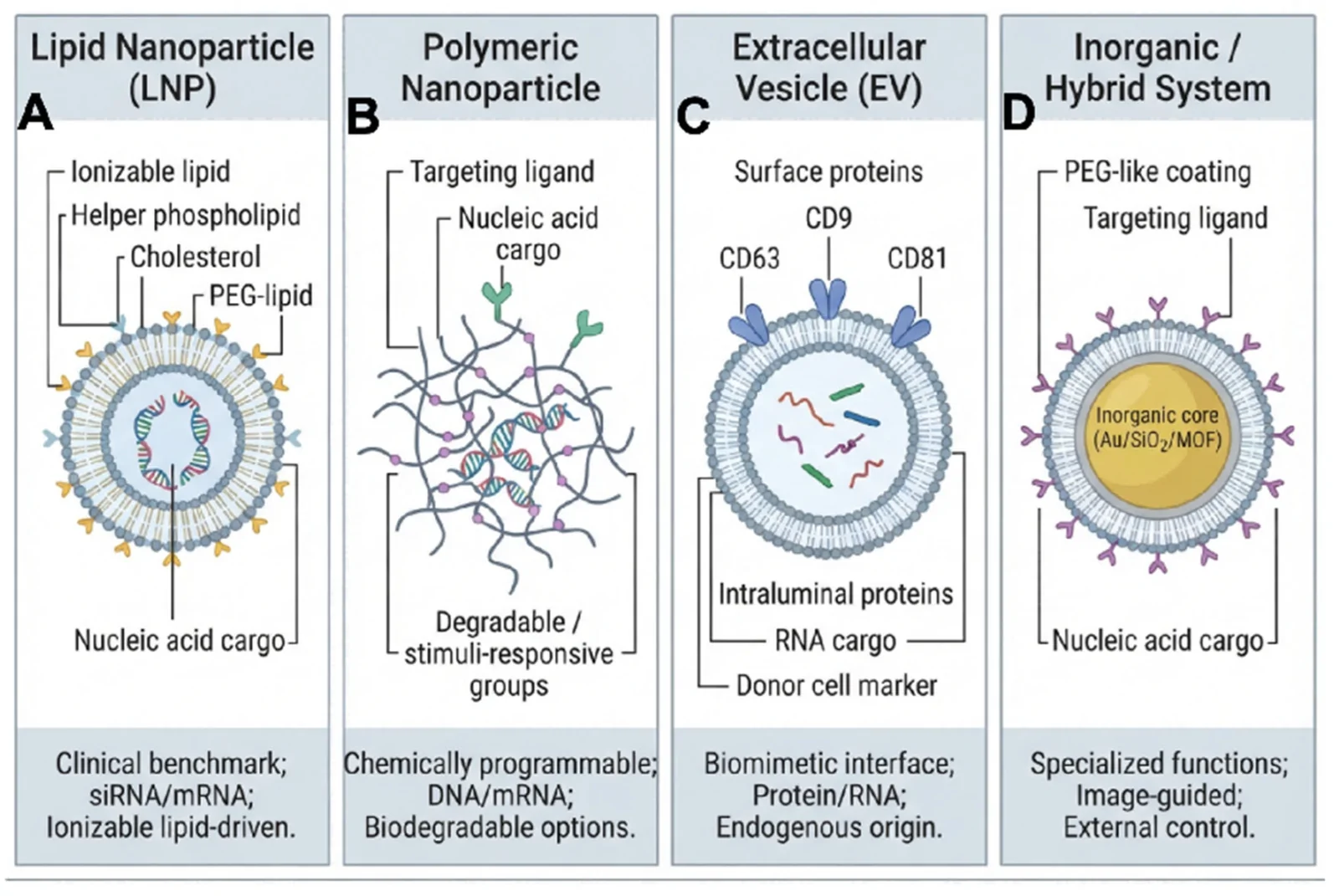
Structural comparison of the four major non-viral vector platforms. (**A**) Lipid nanoparticles (LNPs) consist of an ionizable lipid matrix, helper phospholipids, cholesterol, PEG-lipids, and encapsulated nucleic acid cargo. (**B**) Polymeric nanoparticles are composed of a polymer backbone (e.g., PBAE, PEI) that electrostatically complexes nucleic acids, with optional targeting ligands and stimuli-responsive groups. (**C**) Extracellular vesicles (EVs) feature a natural lipid bilayer membrane with surface proteins (CD9/CD63/CD81), intraluminal proteins, and encapsulated RNA cargo. (**D**) Inorganic/hybrid systems comprise an inorganic core (gold, silica, or MOF) with surface-conjugated nucleic acids, targeting ligands, and PEG coatings.

Figure 1 illustrates the barriers common to all non-viral systems. To address these barriers, four major classes of non-viral vectors have been developed. Their structural architectures are compared in Figure 2.

Several recent reviews have provided valuable syntheses of non-viral gene delivery, including focused comparisons between LNPs and EVs [19], comprehensive overviews of specific platforms [9,10,20], and broad analyses of engineering challenges across viral and non-viral systems [21]. However, a systematic comparison of all four major non-viral platforms—LNPs, polymeric nanoparticles, EVs, and inorganic/hybrid systems—within a unified framework centered on sequential biological barriers and clinical translatability has not been comprehensively addressed. Moreover, while existing reviews have largely treated platforms in isolation or focused on a subset of technologies, the present review explicitly integrates: (i) head-to-head experimental comparisons between platforms, particularly LNPs and EVs; (ii) a comparative framework for evaluating platform performance across clinically relevant dimensions; and (iii) a practical technology selection matrix to guide platform choice based on cargo type, target tissue, and therapeutic context. By bridging mechanistic understanding with translational decision-making, this review aims to provide a resource not only for researchers developing novel delivery systems but also for clinicians and industry scientists selecting appropriate platforms for specific therapeutic applications.

**Literature search methodology.** Literature for this review was identified through systematic searches of PubMed, Web of Science, and Google Scholar using combinations of the following keywords: “non-viral vectors”, “gene delivery”, ‘lipid nanoparticles’, “polymeric nanoparticles”, “extracellular vesicles”, “exosomes”, ‘CRISPR delivery”, “endosomal escape”, and “clinical translation”. The search was limited to articles published in English from January 2015 through July 2026, with emphasis on studies published since 2020. Reference lists of retrieved articles were manually screened for additional relevant studies.

**Distinction between delivery levels.** Throughout this review, we distinguish among several distinct levels of delivery: (i) cellular uptake, which refers to internalization of the vector by target cells; (ii) cytosolic delivery, which requires productive endosomal escape and release of functional cargo into the cytosol; (iii) functional cargo delivery, which requires the delivered cargo (e.g., mRNA, siRNA, or RNP) to retain its biological activity; and (iv) genome editing, which requires the editing machinery to access the nucleus and achieve targeted genomic modification. These distinctions are critical, as high cellular uptake does not necessarily translate to productive cytosolic delivery or functional activity.

## 2. Lipid Nanoparticles: The Most Clinically Mature Non-Viral Platform

### 2.1. LNP Architecture and Functional Components

LNPs usually contain four functional components: an ionizable lipid, a helper phospholipid, cholesterol or a cholesterol analogue, and a PEG-lipid. The ionizable lipid supports cargo encapsulation during acidic formulation and promotes endosomal escape after protonation in the acidic endosomal environment. Helper lipids and cholesterol regulate membrane packing, particle stability, fusion propensity, and intracellular release. PEG-lipids reduce aggregation and influence circulation behavior, but excessive PEGylation can impair cell association and endosomal escape [11,12,13,14,15,16,17,18].

**Figure 3:**
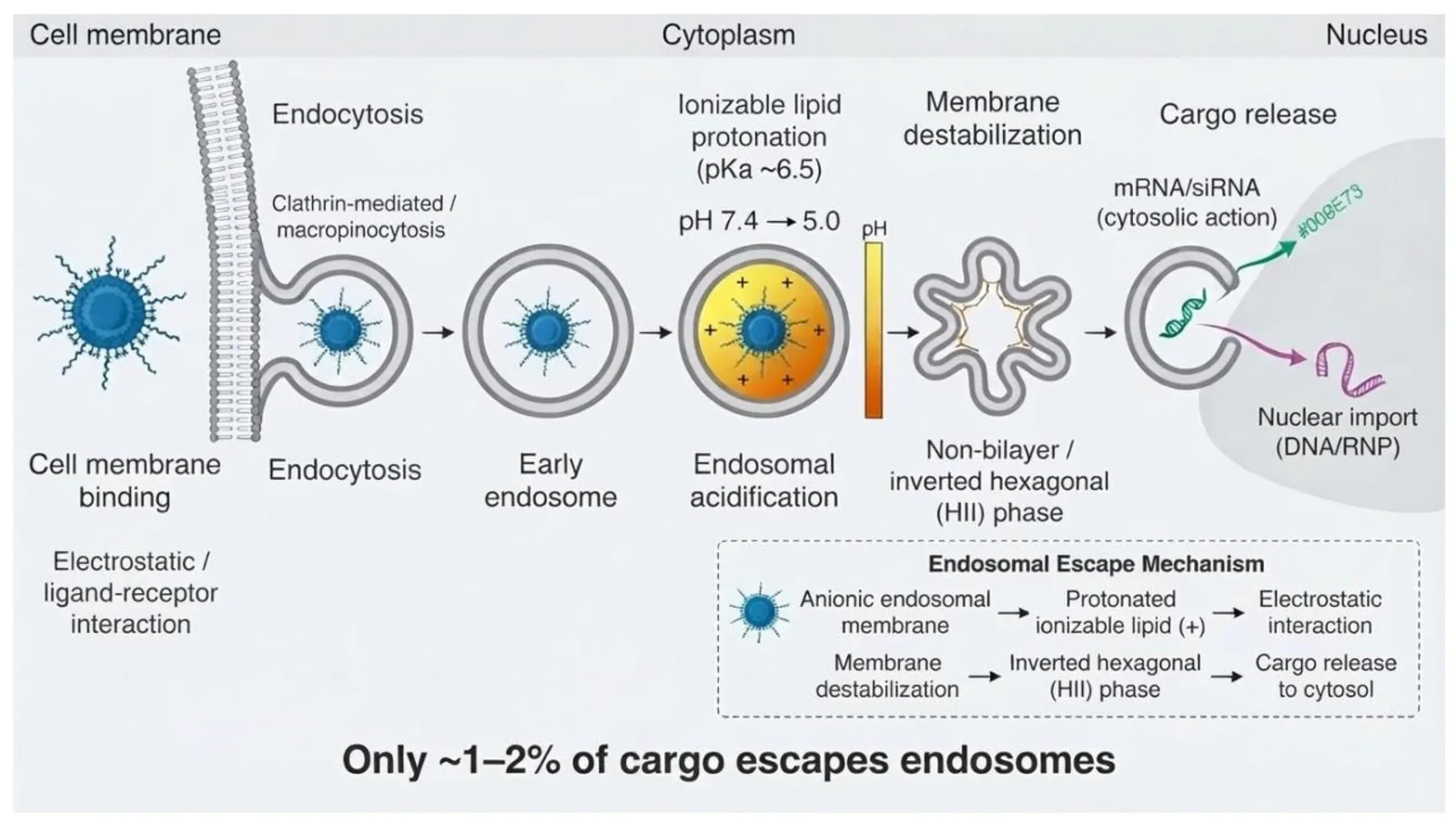
**Mechanisms of LNP-mediated cellular uptake, endosomal escape, and cytosolic cargo release. The six-step process is illustrated:** (1) LNP binding to the cell membrane via electrostatic or ligand-receptor interactions; (2) internalization via clathrin-mediated endocytosis or macropinocytosis; (3) formation of an early endosome containing the LNP; (4) endosomal acidification (pH 7.4 → 5.0) and protonation of the ionizable lipid (pKa~6.5); (5) electrostatic interaction between protonated lipids and anionic endosomal membranes, leading to membrane destabilization and formation of inverted non-bilayer (HII) structures; and (6) release of nucleic acid cargo into the cytosol. Following release, mRNA and siRNA act in the cytosol (translation or RNAi), whereas DNA and RNP cargoes require nuclear import for functional activity. The inset provides a detailed view of the endosomal escape mechanism. The estimated efficiency of endosomal escape is approximately 1–2% of internalized cargo. Color coding: blue, LNP components; green, cytosolic-acting cargo (mRNA/siRNA); purple, nuclear-targeted cargo (DNA/RNP); orange, membrane/intermediate components; red, key bottleneck.

The transition from permanently cationic lipids to ionizable lipids was a major advance in nucleic-acid delivery. Ionizable lipids are usually near-neutral at physiological pH, which may reduce systemic toxicity, but can become positively charged in the endosome, promoting interactions with anionic endosomal lipids and membrane destabilization. Despite these advances, endosomal escape remains one of the major quantitative bottlenecks in nucleic acid medicine. In typical LNP formulations, only a small fraction—estimated at 1–2%—of internalized nucleic acid cargo successfully reaches the cytosol [13,14,15,16,17,18]. The detailed mechanism of LNP-mediated cellular uptake, endosomal acidification, membrane destabilization, and cargo release is illustrated in Figure 3.

Recent strategies include biodegradable ester- or disulfide-containing ionizable lipids, multi-tail lipids, branched lipids, zwitterionic lipids, and ionizable phospholipids. These materials aim to improve cargo release while reducing tissue accumulation and toxicity [18,22,23,24]. For example, membrane-destabilizing ionizable phospholipids have enabled organ-selective mRNA delivery and CRISPR/Cas gene editing by promoting pH-dependent membrane reorganization in endosomes [18]. Representative ionizable lipids that have reached clinical application include DLin-MC3-DMA (used in Onpattro), SM-102 (used in the Moderna COVID-19 vaccine), and ALC-0315 (used in the Pfizer–BioNTech COVID-19 vaccine), each with distinct performance profiles in terms of potency, tolerability, and biodistribution [25,26].

### 2.2. LNP-Mediated Delivery of CRISPR Cargoes

LNPs can deliver CRISPR components in three principal formats: plasmid DNA, Cas mRNA plus sgRNA, and preassembled Cas RNPs. Plasmid DNA is comparatively durable but requires nuclear entry and can sustain editor expression. Cas mRNA/sgRNA avoids DNA nuclear delivery and is compatible with established RNA-LNP formulation processes. Cas RNP delivery offers rapid and transient activity but is more difficult because Cas proteins can aggregate or lose activity during encapsulation and endosomal trafficking [5,6,7,8,9,10,11,12,24,27,28,29,30,31,32].

A landmark study showed that a single systemic administration of biodegradable LNPs containing Cas9 mRNA and chemically modified sgRNA induced persistent Ttr editing in mice, with greater than 97% reduction of serum transthyretin maintained for at least one year [24]. Other studies have demonstrated that biodegradable or redox-responsive LNPs can co-deliver Cas9 mRNA and sgRNA to reduce Pcsk9 expression or enable hepatic editing of disease-relevant genes [27,31].

RNP delivery has become especially attractive because of its transient intracellular activity. Im et al. showed that LNP formulation conditions must be adjusted for protein-containing cargoes: near-neutral or mildly acidic preparation conditions can better preserve Cas9 activity than strongly acidic conditions commonly used for RNA-LNP assembly [30]. More recently, optimized LNP formulations achieved enhanced in vivo delivery of base-editor and prime-editor RNPs, demonstrating that transient RNP delivery can support editing while potentially limiting prolonged editor exposure [32].

### 2.3. Extrahepatic Delivery and Organ Selectivity

Systemically administered LNPs predominantly exhibit liver tropism—with over 90% of the injected dose accumulating in the liver within one hour for many formulations—because of hepatic vascular anatomy and interactions with serum proteins such as apolipoprotein E. This characteristic is advantageous for liver diseases but constrains application to extrahepatic tissues [14,16,33]. Consequently, a central objective in LNP engineering is to achieve delivery beyond hepatocytes and toward defined cell populations in the lung, spleen, bone marrow, central nervous system, muscle, tumor microenvironment, and immune system [10,11,33,34,35,36,37,38,39,40,41,42,43].

Selective organ targeting (SORT) demonstrated that addition of a supplementary lipid component can shift LNP biodistribution toward the lung, spleen, or liver and support tissue-specific mRNA delivery and CRISPR editing [22]. Chemical composition alone can also influence cell tropism. In vivo directed-evolution approaches and barcode-based screens have identified LNPs capable of delivering RNA to bone-marrow endothelial cells or defined immune-cell populations [38,40]. Recent work has further shown that liposomal LNP systems—achieved by increasing the phospholipid-to-ionizable lipid ratio—exhibit extended circulation times and enhanced extrahepatic transfection, attributed to reduced plasma protein adsorption [6,18].

Pulmonary delivery is an especially important example. Inhalable LNPs generated from combinatorial biodegradable ionizable-lipid libraries have enabled repeated pulmonary mRNA delivery and lung epithelial genome editing [34]. More recent studies have reported lung-tropic LNP formulations that edit endothelial and epithelial cells while limiting off-target liver editing [35]. In cystic fibrosis models, LNP optimization together with mucus-barrier modulation improved functional gene editing in increasingly complex bronchial tissue systems, emphasizing the importance of physiologically relevant models for evaluating delivery [36].

### 2.4. Clinical Translation of LNP-Based Therapies

The clinical utility of LNP-mediated delivery is exemplified by multiple approved products and advancing programs. The siRNA therapeutic patisiran (Onpattro), delivered via LNP, was the first FDA-approved RNAi therapeutic for hereditary transthyretin-mediated amyloidosis [44], while the COVID-19 mRNA vaccines (Comirnaty and Spikevax) validated the LNP platform at unprecedented scale [25,26].

Preclinical studies of the PCSK9 base-editing candidate VERVE-101 in nonhuman primates demonstrated durable PCSK9 and LDL-C reductions of up to 83% and 69%, respectively, supporting the clinical translation of this approach [45].

For genome editing, NTLA-2001 (nexiguran ziclumeran), targeting hepatic TTR, was the first systemically administered CRISPR therapy to enter clinical testing, demonstrating durable reductions in circulating transthyretin after a single infusion [46]. Following a clinical hold imposed in October 2025 due to a serious adverse event, the FDA lifted the hold on the MAGNITUDE-2 (ATTRv-PN) Phase 3 trial in January 2026 and on the MAGNITUDE (ATTR-CM) Phase 3 trial in March 2026, allowing both trials to proceed [1,46]. NTLA-2002 (lonvoguran ziclumeran), targeting KLKB1 for hereditary angioedema, met its primary and key secondary endpoints in the Phase 3 HAELO trial, and a Biologics License Application is under FDA review [1,2].

Beyond Intellia’s programs, additional LNP-based in vivo editing therapies have entered clinical development. YOLT-101, an adenine base-editing therapy delivered via GalNAc-modified LNPs targeting PCSK9 for heterozygous familial hypercholesterolemia, has reported Phase 1 results in Nature Medicine, showing PCSK9 reduction of 74.4% and LDL-C reduction of 52.3% at 24 weeks in the 0.6 mg/kg cohort [47]. CS-121, an LNP-delivered base-editing therapy targeting APOC3 for familial chylomicronemia syndrome, is in Phase 1 clinical development (NCT07176923) [48]. CTX310, a lipid nanoparticle formulation delivering Cas9 mRNA and guide RNA targeting ANGPTL3, has entered Phase 1 trials for refractory dyslipidemias (NCT07491172), with early results published in the New England Journal of Medicine [49].

These programs illustrate the advantage of transient RNA-mediated editing: the editing machinery is present only temporarily, whereas the genomic modification may persist. Nevertheless, clinical translation requires long-term follow-up for potential off-target editing, immune activation, infusion reactions, liver toxicity, and durability of therapeutic benefit [3,11,46,50]. Importantly, off-target biodistribution—accumulation of vectors in non-target organs—should be distinguished from off-target genome editing—unintended genomic modifications at non-target sites—as the former may cause tissue toxicity without necessarily resulting in the latter [3,50].

## 3. Polymeric Nanoparticles: Programmable Chemistry and Controlled Intracellular Release

### 3.1. Strengths and Limitations of Polymeric Vectors

Polymeric carriers offer extensive molecular-design flexibility. Their charge density, branching architecture, molecular weight, hydrophobicity, degradability, buffering capacity, and surface ligands can be tuned for specific cargoes or tissues. Common materials include polyethyleneimine, poly(β-amino ester)s (PBAEs), poly(amidoamine)s, chitosan derivatives, dendrimers, peptide–polymer conjugates, and polymer–lipid hybrids [20,51,52,53,54].

The classical advantage of cationic polymers is nucleic acid condensation through electrostatic interactions. However, strong and persistent positive charge can also lead to hemolysis, complement activation, cytotoxicity, non-specific uptake, and poor systemic compatibility. Thus, modern polymeric-vector research increasingly emphasizes biodegradable backbones, charge-shielding strategies, acid-labile or redox-responsive linkages, and cargo release after internalization [20,51,52,53,54,55,56].

### 3.2. Poly(β-amino ester)s and Branched Polymer Architectures

PBAEs are among the most widely investigated degradable polymeric platforms. Their synthesis is modular, allowing substantial variation in backbone chemistry, terminal groups, and branching. Highly branched PBAEs have demonstrated substantially improved nucleic-acid delivery compared with related linear materials, likely because branching affects condensation, particle stability, uptake, and intracellular release [53,54].

Recent studies have further shown that tri- and tetrafunctional branching can produce small, monodisperse polymeric nanoparticles with efficient DNA, mRNA, and siRNA delivery [57]. High-content screening platforms integrating uptake and Galectin-8-based endosomal-disruption measurements have also shown that productive delivery cannot be predicted simply from particle size or zeta potential [58].

### 3.3. Polymer-Mediated CRISPR Delivery

Polymeric vectors can deliver plasmid DNA, Cas mRNA/sgRNA, or Cas RNPs. Their modularity is particularly attractive for RNP delivery, because they can be engineered to interact with both protein and nucleic-acid components. Guanidinobenzol-rich polymers, for example, have been developed to facilitate CRISPR RNP delivery across sequential barriers including cell entry and endosomal escape [59].

For DNA-based delivery, nuclear transport remains crucial. Chitosan–plasmid nanoparticles modified with karyophilic peptides illustrate the importance of engineering not only cell entry but also nuclear localization [60,61]. Peptide vectors such as PepFect14 can also form stable nucleic-acid nanoparticles and facilitate delivery in difficult-to-transfect cells [62].

### 3.4. Stimuli-Responsive and Hybrid Polymeric Systems

Stimuli-responsive polymers may improve therapeutic selectivity by releasing cargo in response to pH, redox state, enzyme activity, light, or other local signals. Such systems are particularly appealing for tumors and inflamed tissues, although their biological responsiveness and safety must be established under clinically relevant conditions [56,57,63].

Hybrid lipid–polymer nanoparticles may combine the high nucleic-acid encapsulation and membrane interactions of lipid systems with the structural stability and functional versatility of polymers. These approaches may be especially relevant for large RNA cargoes or RNPs but increase product complexity and regulatory burden [20,64,65].

## 4. Inorganic and Specialized Nanocarriers

Inorganic materials such as gold nanoparticles, magnetic nanoparticles, mesoporous silica nanoparticles, calcium phosphate systems, and metal–organic frameworks provide features less accessible to conventional LNPs, including imaging contrast, magnetic guidance, photothermal responsiveness, rigid porous structures, and externally triggered release [66,67,68,69,70,71,72,73].

CRISPR-Gold, composed of gold nanoparticles, DNA, Cas RNP, donor DNA, and an endosomal-disruptive polymer, demonstrated non-viral homology-directed repair in vivo and correction of a Duchenne muscular dystrophy model after local administration [67]. Gold- and silica-based systems can also support photothermal or stimulus-responsive release, but long-term biodistribution and potential inorganic-material accumulation remain important translational issues [68,69,70,71,72,73].

Inorganic systems should therefore be considered specialized rather than universal vectors. Their strongest use cases may involve local administration, image-guided treatment, externally triggered release, combination therapy, or applications in which their structural or physical properties provide a clear advantage over biodegradable lipid and polymer systems [66,67,68,69,70,71,72,73].

## 5. Extracellular Vesicles and Biomimetic Delivery Systems

### 5.1. Biological Rationale for EV-Based Delivery

EVs are cell-derived membrane particles that transfer proteins, lipids, and nucleic acids between cells. Their endogenous origin, membrane composition, and potential tissue-interaction properties have motivated their development as delivery vehicles [19,73,74,75,76]. However, EVs are heterogeneous populations rather than a single uniform product category. Their composition and activity depend on the donor cell, culture conditions, isolation method, loading strategy, and route of administration [77,78,79,80,81].

The potential advantages of EVs include biocompatibility, ability to transport diverse biomolecules, and opportunities for donor-cell engineering or surface modification. Their disadvantages include variable cargo loading, limited yield, complex purification, uncertain biodistribution, and major analytical and manufacturing challenges [19,74,75,76,77,78,79,80,81,82,83,84,85,86].

A critical point is that the “natural” origin of EVs should not be equated with inherent safety or superior targeting. Direct comparative studies have revealed that EV performance is often comparable to—or in some cases inferior to—synthetic LNPs when evaluated under standardized conditions, and the purported tissue-homing properties of EVs frequently derive from the donor cell type rather than from a generalizable EV property [19]. The field must therefore move beyond claims of innate EV superiority and instead focus on specific use cases where the EV membrane interface provides a demonstrable advantage.

### 5.2. EV-Mediated Delivery of RNA and CRISPR Systems

EVs have been engineered to deliver siRNA, miRNA, antisense oligonucleotides, mRNA, plasmid DNA, Cas9 RNPs, and RNA-editing systems [74,75,76,87,88,89,90,91,92,93,94,95,96]. Red blood cell-derived EVs are particularly interesting because they can be produced from an enucleated cell source and have delivered antisense oligonucleotides, Cas9 mRNA, and guide RNAs in preclinical models [89].

Engineered EVs carrying Cas9 RNPs targeting Pcsk9 have achieved functional editing in primary mouse hepatocytes [87]. Other strategies include CD63-linked cargo-loading systems, RNA aptamer–protein interactions, GFP nanobody approaches, and fusogenic components intended to improve cytosolic release [85,90,93].

A key principle emerging from these studies is that loading alone is insufficient. Functional delivery depends on a balance between efficient capture of cargo during EV formation and efficient release after entry into recipient cells. Excessively tight interactions between cargo and EV-loading modules may impair intracellular cargo release [93].

### 5.3. EV Engineering, Hybrid Vesicles, and Quality Attributes

EV engineering can occur at the donor-cell level, during EV biogenesis, after isolation, or through fusion with synthetic particles. Approaches include surface-ligand display, cationization, electroporation, pH-gradient loading, lipid insertion, and formation of EV–liposome hybrids [80,84,88,94,95,96].

Cationized EVs can improve loading of plasmid DNA, mRNA, and siRNA, but increased surface charge may affect protein adsorption, toxicity, and in vivo clearance [88]. EV–liposome hybrids may increase loading capacity while retaining elements of the biological membrane interface [80]. These systems are promising but require careful characterization of membrane composition, cargo location, fusion efficiency, and batch consistency.

The Minimal Information for Studies of Extracellular Vesicles (MISEV2023) guidelines and related analytical frameworks should be integrated into EV-based gene-delivery studies [4,77,78,79,80,81,82,83]. Essential quality attributes include source-cell identity, particle concentration, size distribution, marker profile, purity, cargo quantity, sterility, potency, residual impurities, and storage stability [77,78,79,80,81,82,83]. Critically, EV heterogeneity is not merely a challenge to be overcome but a fundamental property that must be characterized and managed rather than eliminated. The goal should be to define acceptable ranges of variability rather than to pursue perfect homogeneity.

### 5.4. EV-Specific Translational Barriers

Beyond the general challenges of heterogeneity and manufacturing complexity, several EV-specific barriers warrant dedicated consideration.

**Engineering feasibility and purification-associated contaminants.** Unlike synthetic LNPs, which are chemically defined, EVs are produced biologically, requiring extensive purification to remove co-isolated contaminants such as protein aggregates, lipoproteins, and non-EV particles. Purification method dramatically influences the purity and functional integrity of EV preparations, with ultracentrifugation, size-exclusion chromatography, and immunoaffinity capture yielding preparations with distinct purity profiles and biological activities [97].

**Donor cell-dependent variability.** The composition and functional properties of EVs vary substantially depending on the producer cell type, culture conditions, and passage number. This donor cell dependency introduces batch-to-batch variability that is not encountered with chemically defined synthetic platforms, complicating reproducibility and regulatory qualification [98].

**Biodistribution and off-target accumulation.** The biodistribution of EVs is influenced by the source cell type and surface protein composition, with significant implications for therapeutic applications. Tumor-derived EVs, for example, can redirect therapeutic nanoparticles to hepatic Kupffer cells, thereby reducing tumoral accumulation [19,86].

**Repeat-dose immunogenicity.** While EVs are often assumed to be immunologically neutral, repeated administration of xenogeneic or allogeneic EVs can elicit immune responses that compromise efficacy and safety. The immunogenicity of EV preparations differs depending on the origin of manufacturing cells, the presence of donor cell-derived surface proteins, and the nucleic acid content of the EVs [98].

## 6. Comparative Assessment of Non-Viral Platforms

### 6.1. Overall Platform Comparison

LNPs are currently the practical benchmark for systemic nucleic-acid delivery because they combine high encapsulation efficiency, scalable manufacturing, and extensive clinical experience. The clinical approvals of patisiran and the mRNA COVID-19 vaccines have established a clear regulatory pathway for LNP-based products [11,12,13,14,15,16,99,100,101,102,103,104,105,106]. Polymeric vectors may be advantageous where biodegradable, locally administered, or highly cargo-specific formulations are required [20,51,52,53,54,55,56,57,58,59,104,105,106,107]. EVs may offer differentiated value when their biological interface, endogenous targeting behavior, or ability to carry protein complexes clearly outweighs their manufacturing complexity [19,74,75,76,77,78,79,80,81,82,83,84,85,86].

A comprehensive comparative summary of these four platforms is provided in Table 2, which highlights their respective strengths, limitations, and best-developed applications based on the literature cited throughout this section.

#### Direct Head-to-Head Comparisons Between LNPs and EVs

Several recent studies have directly compared LNP and EV platforms for nucleic acid delivery under controlled conditions. Murphy et al. demonstrated that while LNPs achieved higher overall cellular uptake, EVs exhibited distinct intracellular trafficking kinetics with a greater proportion of cargo escaping the endolysosomal pathway [108]. Conversely, Zhuo et al. showed that EV-mediated delivery resulted in lower immunogenicity in vivo but significantly reduced cargo loading efficiency compared to LNPs [109]. Bader et al. systematically compared the two platforms across biodistribution, cellular uptake, and manufacturing parameters, concluding that neither platform is universally superior—rather, the optimal choice depends on cargo type, administration route, and therapeutic context [19]. These head-to-head studies underscore that the relative performance of LNPs and EVs is highly context-dependent and that the two platforms should be viewed as complementary rather than competing tools in the gene delivery toolbox (Table 3).

### 6.2. Comparison by Delivery Barrier

Circulation stability and biodistribution. The biodistribution of LNPs is governed by lipid composition, particle size, PEGylation degree, and the protein corona. Following intravenous administration, most LNPs exhibit pronounced hepatic accumulation, with over 90% of the injected dose cleared to the liver within one hour for many formulations [14,16,33]. Polymer biodistribution and hemocompatibility are highly dependent on charge density and degradation characteristics. Highly cationic polymers may be rapidly cleared and can cause complement activation [20,52]. EV biodistribution is influenced by the cell of origin, surface protein composition, administration route, and host immune status; the intrinsic heterogeneity of EV preparations translates to variable biodistribution profiles [19,77].

Target tissue accumulation and cell-type specificity. A critical future objective is the transition from “organ targeting” to “cell-type-specific delivery.” Achieving organ-level accumulation—even in the liver or lungs—is insufficient to ensure therapeutic efficacy if the payload is taken up by non-target cells within that organ. Kupffer cells in the liver, phagocytic cells in the spleen, and stromal cells in tumors can sequester nanoparticles intended for parenchymal or tumor cells [11,38,39,40,41,42,43]. Strategies to improve cellular selectivity include ligand conjugation (antibodies, peptides, aptamers), lipid structure engineering, protein corona modulation, and local administration [11,38,39,40,41,42,43,115,116,117,118].

**Endosomal escape: the common rate-limiting step.** The vast majority of nanoparticles that enter cells are trafficked through the endolysosomal pathway, and only a small fraction—estimated at approximately 1–2% for typical LNP formulations—successfully escape to the cytosol [15,16,17,18]. However, this estimate should not be generalized uniformly to all non-viral systems. EVs may utilize alternative endosomal escape mechanisms, including membrane fusion and endosomal back-fusion [119], which could result in distinct and potentially more efficient cargo release profiles. The precise efficiency of EV-mediated cytosolic delivery remains less well characterized due to methodological challenges in quantifying functional cargo release from EVs, but emerging evidence suggests that the entry and trafficking pathways of EVs diverge substantially from those of synthetic LNPs [19]. Therefore, endosomal escape efficiency should be evaluated on a platform-specific basis rather than treated as a universal limitation across all non-viral vectors.

Manufacturing, scalability, and regulatory acceptability. LNPs benefit from a relatively clear manufacturing and quality control foundation, with established processes for lipid synthesis, nanoparticle formulation (typically via microfluidics or T-junction mixing), and characterization [99,100,101,102,103]. Polymeric systems require control over molecular weight, polydispersity, degree of substitution, and degradation behavior—parameters that can vary substantially with synthesis conditions [20,47,48,49,50,51,52,53,54,55,56,57,58,59,104,105,106,107,108]. EVs face the most significant manufacturing challenges: the development of standardized definitions for source material, purity, particle number, cargo content, potency, and long-term storage stability is essential for the field to advance beyond proof-of-concept studies [77,78,79,80,81,82,83,84,85,86].

### 6.3. GalNAc Conjugation: A Complementary Clinical Paradigm

While the primary focus of this review is on nanoparticle-based delivery systems, a brief discussion of GalNAc conjugation is included here because it represents a complementary clinically validated paradigm for liver-targeted nucleic acid delivery that has achieved regulatory approval without requiring nanoparticle formulation. This conjugation strategy is not a nanoparticle platform per se and is limited to hepatocyte targeting via ASGPR, but it is included to provide a complete picture of clinically available non-viral nucleic acid delivery strategies.

While LNPs represent the dominant platform for systemic nucleic acid delivery, *N*-acetylgalactosamine (GalNAc) conjugation offers a complementary clinically validated approach for liver-targeted siRNA delivery. GalNAc ligands bind with high affinity to the asialoglycoprotein receptor (ASGPR), which is abundantly and selectively expressed on hepatocytes. This conjugation strategy enables potent and durable gene silencing with subcutaneous administration and has been clinically validated in multiple approved products, including inclisiran (Leqvio) for PCSK9 silencing in hypercholesterolemia, givosiran for acute hepatic porphyria, lumasiran for primary hyperoxaluria type 1, and vutrisiran for hereditary transthyretin-mediated amyloidosis [3]. Unlike LNP formulations, GalNAc conjugates do not require complex nanoparticle assembly and can be manufactured as defined chemical entities, substantially simplifying regulatory and quality control requirements. However, GalNAc conjugation is limited to hepatocyte targeting via ASGPR and is not readily adaptable to extrahepatic tissues or to mRNA and CRISPR cargoes that require endosomal escape mechanisms beyond receptor-mediated uptake. Thus, GalNAc conjugates and LNPs should be viewed as complementary rather than competing platforms, each suited to different cargo types and therapeutic indications (Table 4).

## 7. Future Directions

### 7.1. From Organ Targeting to Cell-Type-Selective Delivery

Organ-level accumulation is insufficient for many therapeutic applications. For example, liver uptake does not guarantee hepatocyte specificity, tumor accumulation does not guarantee uptake by malignant cells, and lung delivery does not guarantee access to the disease-relevant epithelial or endothelial cell population. Future delivery systems should therefore be evaluated by cell-resolved biodistribution and functional activity rather than bulk tissue fluorescence alone [31,33,38,39,40,41,42,43,44,120].

Surface ligands, antibodies, peptides, aptamers, biomolecular-corona modulation, and local administration may improve targeting but also increase formulation complexity, cost, and potential immunogenicity. Therefore, targeting strategies must demonstrate a meaningful improvement in functional therapeutic index rather than merely altered biodistribution [11,38,39,40,41,42,43].

### 7.2. Endosomal Escape as a Central Engineering Objective

Endosomal entrapment limits the productive cytosolic delivery of almost all nanoparticle classes. Future development should prioritize quantitative assays for cytosolic release and endosomal disruption, including approaches that distinguish total cell association from functional delivery [17,53,59,91].

Potential strategies include optimization of ionizable-lipid pKa, incorporation of fusogenic or membrane-disruptive motifs, pH-responsive polymers, redox-responsive linkers, endosomolytic peptides, and transient external stimuli such as light or ultrasound [17,18,56,91,115]. However, improving endosomal escape must not be achieved at the expense of unacceptable membrane toxicity or systemic inflammation.

### 7.3. Delivery of Base Editors, Prime Editors, and Large Cargoes

Base editors and prime editors can avoid some limitations of double-strand-break-based editing but are larger and more complex than Cas9 nucleases. Their delivery may require optimized mRNA-LNP systems, RNP formulations, split-editor strategies, dual-particle delivery, or synchronized sequential delivery [10,11,32,104,120,121,122].

The optimal vector for a genome editor should be selected according to cargo size, required duration of activity, target-cell type, editing endpoint, and safety profile. A carrier optimized for hepatic Cas9 knockout should not be assumed to be optimal for base editing, prime editing, homology-directed repair, or extrahepatic delivery [5,6,7,8,9,10,11,29,30,31,32].

### 7.4. Repeat Dosing: Challenges and Strategies

Repeat dosing of non-viral vectors presents unique challenges that are often overlooked in preclinical studies focused on single-administration efficacy. For LNPs, anti-PEG antibodies can develop after initial exposure, leading to accelerated blood clearance and reduced efficacy upon subsequent dosing [99]. Lipid accumulation in phagocytic cells and potential hepatotoxicity also raise concerns for chronic or repeat-administration regimens. Strategies to address these challenges include the use of alternative PEG-lipids with lower immunogenicity, exchangeable PEG-lipids that desorb from particles in circulation, non-PEGylated formulations, and the incorporation of biodegradable lipids that are cleared more rapidly [99,100,101,102,103,104]. For polymer systems, cumulative toxicity from non-degradable materials and immune responses to polymer backbones must be carefully evaluated. For EVs, the immunogenicity of repeated administrations—particularly from xenogeneic or allogeneic sources—remains poorly characterized [77,78,79,80,81,82,83]. Future development should therefore incorporate repeat-dosing studies at the preclinical stage and consider immunogenicity as a primary rather than secondary design parameter.

### 7.5. Manufacturing, Quality Control, and Regulatory Translation

The clinical success of a non-viral vector requires simultaneous optimization of delivery potency, safety, and manufacturability. For LNPs, critical quality attributes include particle size, polydispersity, RNA integrity, encapsulation efficiency, lipid composition, potency, sterility, endotoxin, and stability [99,100,101,102,103]. Novel ionizable-lipid excipients may require extensive chemistry, manufacturing, and controls packages because their identity and function are inseparable from product performance [99].

**Figure 4:**
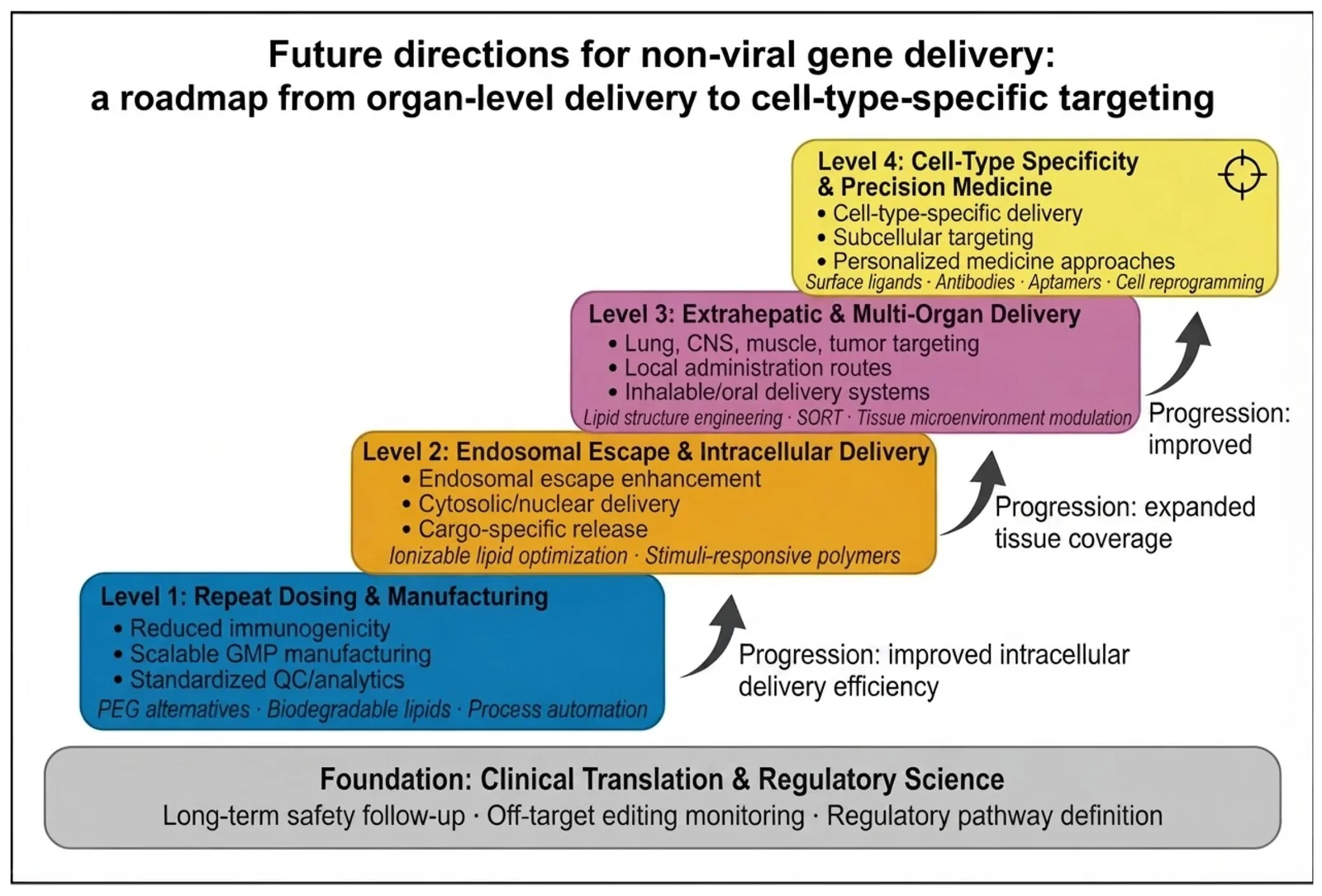
Future directions for non-viral gene delivery: a roadmap from organ-level delivery to cell-type-specific targeting. Four hierarchical levels of advancement are illustrated: (Level 1) repeat dosing and scalable manufacturing, addressing immunogenicity and quality control; (Level 2) endosomal escape and intracellular delivery, focusing on cytosolic and nuclear cargo release; (Level 3) extrahepatic and multi-organ delivery, expanding the range of target tissues beyond the liver; and (Level 4) cell-type specificity and precision medicine, representing the ultimate goal of delivering the right cargo to the right cell population. The foundation layer (clinical translation and regulatory science) supports all higher-level advancements. Key technologies enabling each level are indicated.

For polymeric systems, molecular-weight distribution, branching, substitution degree, residual monomer content, and degradation products must be carefully controlled [20,51,52,53,54,55,56,57,58,59,104,105,106,107]. For EVs, the challenge is even greater: standardized source-cell culture, isolation, characterization, potency testing, and storage methods are essential before broad clinical implementation [77,78,79,80,81,82,83,84,85,86], as illustrated in Figure 4.

## 8. Conclusions

Non-viral vectors have progressed from lower-efficiency alternatives to viral delivery systems into central technologies for RNA medicines and in vivo genome editing. LNPs currently have the strongest clinical foundation, particularly for hepatic delivery, supported by approved siRNA therapeutics, mRNA vaccines, and advancing Phase 3 CRISPR editing programs. However, LNPs must overcome extrahepatic targeting and endosomal-escape limitations to reach their full therapeutic potential. Polymeric vectors remain valuable because of their chemical versatility and potential for tailored intracellular engineering, though their clinical translation has been constrained by toxicity and reproducibility challenges. EVs and biomimetic hybrids offer promising biological interfaces for nucleic-acid and protein delivery but require major advances in loading efficiency, product standardization, and scalable manufacturing before they can compete with synthetic platforms in the clinic.

The next phase of the field will be defined by the ability to deliver the right cargo to the right cell population, achieve productive intracellular release, minimize immune and off-target effects, and manufacture a reproducible product at clinically relevant scale. Critically, platform selection should be driven by the specific requirements of the therapeutic application—cargo type, target tissue, required duration of activity, and safety profile—rather than by technological fashion. No single non-viral platform will be universally optimal; the future lies in a diversified toolkit of vectors matched to distinct clinical scenarios. The most pressing unsolved questions include: How can endosomal escape efficiency be improved from the current ~1–2% to a therapeutically meaningful level? How can cell-type-specific delivery be achieved without compromising manufacturability? And how can the immunogenicity of repeat dosing be managed across different vector platforms? Addressing these questions will require not only advances in materials chemistry but also deeper understanding of nanoparticle–biology interactions at the molecular and cellular levels.

## Figures and Tables

**Table 1:** Cargo formats and their distinct delivery requirements.

Cargo Format	Site of Action	Key Delivery Requirements	Preferred Vector Platforms
Plasmid DNA	Nucleus	Cell entry, endosomal escape, nuclear import	Polymers, liposomes, some LNPs
siRNA/ASO	Cytosol	Endosomal escape, cytosolic release	LNP, GalNAc conjugates
mRNA	Cytosol	Endosomal escape, cytosolic release, stability	LNP, polymeric nanoparticles
Cas9 mRNA + sgRNA	Cytosol (translation) + nucleus (editing)	Co-delivery, temporal matching of components	LNP
Cas RNP	Nucleus	Protein stability, co-delivery, nuclear access	Optimized LNP, EV, polymer systems
Base editor/Prime editor	Nucleus	Larger cargo, precise delivery, transient activity	mRNA-LNP or RNP-LNP

**Table 2:** Comparative assessment of non-viral vector platforms.

Parameter	Lipid Nanoparticles	Polymeric Nanoparticles	Extracellular Vesicles	Inorganic/Hybrid Systems
Clinical maturity	Highest (approved siRNA, mRNA vaccines; Phase 3 editing)	Intermediate (limited clinical translation)	Early (preclinical to early clinical)	Mostly preclinical
Typical cargoes	siRNA, mRNA, sgRNA, plasmid DNA, RNP	DNA, RNA, proteins, RNP	RNA, proteins, RNP, selected DNA cargo	DNA, RNA, RNP, combination cargoes
Major advantage	Established clinical precedent and scalable manufacturing	Molecular programmability and degradability	Biological membrane interface and biomimicry	Imaging, external control, specialized release
Major limitation	Endosomal escape and liver bias	Cationic toxicity and variable in vivo performance	Heterogeneity and manufacturing complexity	Long-term safety and biodegradation
Best-developed applications	Hepatic diseases, infectious diseases (vaccines), and in vivo editing	Local/topical delivery and ex vivo cell engineering	Protein/RNP delivery and biomimetic applications	Local, image-guided, or stimulus-responsive delivery
Key references	[11,12,13,14,15,16,17,18,19,20,21,22,23,24,25,26,27,28,29,30,31,32,33,34,35,36,37,38,39,40,41]	[20,51,52,53,54,55,56,57,58,59,104,105,106,107]	[19,74,75,76,77,78,79,80,81,82,83,84,85,86]	[66,67,68,69,70,71,72,73]

**Table 3:** Head-to-head experimental comparisons between LNP and EV platforms.

Reference	Cargo	Key Finding	Implication
Murphy et al. [108]	mRNA	LNPs: higher uptake; EVs: favorable intracellular release	Platform choice depends on delivery barrier
Zhuo et al. [109]	siRNA	EVs: lower immunogenicity; LNPs: higher loading	Trade-off between safety and potency
Bader et al. [19]	Multiple	Systematic comparison across biodistribution, uptake, manufacturing	Complementarity rather than superiority
Ma et al. [21]	Cas9 RNP	EVs: better protein delivery; LNPs: superior nucleic acid delivery	Cargo-specific optimization
Liu et al. [110]	IL-12 mRNA (lung cancer)	Inhalable IL-12 mRNA-EVs suppressed lung tumors and promoted systemic immunity with lower toxicity than LNPs	EV advantages translate to therapeutic benefit in cancer immunotherapy
Chiang et al. [111]	Therapeutic RNA (PDAC)	Dual-targeted EVs effectively suppressed large PDAC tumors	EVs can overcome solid tumor barriers
Ma et al. [112]	VEGF-A/BMP-2 mRNA (bone regeneration)	EVs enabled coupled angiogenic-osteogenic repair	EVs suitable for multi-cargo delivery in tissue regeneration
You et al. [113]	VEGF-A mRNA (cardiac repair)	EVs: lower immunogenicity vs. AAV and LNP	EVs may offer safety advantages for cardiac delivery
Popowski et al. [114]	mRNA/protein (lung delivery)	Inhalable lung-derived EVs showed superior pulmonary biodistribution and retention vs. LNPs	EVs offer platform-level advantages for pulmonary delivery

**Table 4:** Technology Selection Framework.

Clinical/Research Scenario	Recommended Cargo	Preferred Platform	Evidence Level
Hepatic gene silencing	siRNA	LNP or GalNAc conjugate	Approved (clinical)
Hepatic gene editing	mRNA/sgRNA or RNP	LNP	Phase 3
In vivo editing requiring minimal editor exposure	Cas RNP	Optimized LNP or engineered EV	Preclinical/early clinical
Local plasmid DNA delivery	Plasmid DNA	Polymer or chitosan systems	Preclinical
Pulmonary genetic diseases	mRNA/sgRNA	Inhaled/locally administered LNP	Preclinical
Complex biological barriers or biomimetic delivery	RNA, protein, RNP	Engineered EV or EV-hybrid systems	Preclinical
Tumor-targeted delivery	mRNA, siRNA, editors	Targeted LNP, polymer, or combination	Preclinical/early clinical

## Data Availability

No new datasets were generated in this review. All data discussed are available from the cited primary literature and clinical trial registries. Detailed literature search strategies are provided in the Introduction. The comparative assessment framework is described in Section 6.1 and Table 2.
